# Crossbreeding experiment on Indonesian local rabbits: the heterosis effect on growth performance

**DOI:** 10.5194/aab-67-231-2024

**Published:** 2024-05-24

**Authors:** Asep Setiaji, Dela Ayu Lestari, Nuruliarizki Shinta Pandupuspitasari, Ikania Agusetyaningsih, Sutopo Sutopo, Edy Kurnianto

**Affiliations:** Department of Animal Science, Faculty of Animal and Agricultural Sciences, Universitas Diponegoro, Semarang 50275, Central Java, Indonesia

## Abstract

The study aims to investigate the heterosis effect of crossing two imported rabbits with local rabbits on growth characteristics and performance using a nonlinear regression model. The study utilized three rabbit breeds: Flemish Giant rabbits (F), Rex rabbits (R), and Indonesian local rabbits (L). Selective breeding consisted of three breeds: F (FF), R (RR), and L (LL). Two crosses were formed between the male ancestors of L and the respective female ancestors of F (LF) and R (LR). Each offspring's body weight (BW) was measured every 3 d starting from birth. FF exhibited the highest BW among purebred animals. The LF crossbreed had a greater estimated mature weight compared with purebred animals. The predicted mature live weight of the asymptotic value for LR animals was higher than for LL rabbits but lower than for RR rabbits. The heterosis effect was lower when crossing L and F animals compared with crossing L and R animals. The average heterosis impact values were 4.68 for LF and 15.32 for LR. LF rabbits showed superior heterosis effects when the growth parameter and inflection point were determined using a logistic model. This study emphasizes the use of strategic breeding to optimize rabbit growth and performance by offering detailed insights into growth dynamics and heterosis effects in different crossbreeding situations.

## 1 Introduction

Crossbreeding has been shown to be a potent technique for enhancing desired traits in domesticated animals, with the goal of efficient and sustainable livestock production (Naskar et al., 2012; Colditz and Hine, 2016). Due, in great part, to their rapid reproduction and adaptability, rabbits are considered to be a high-quality source of protein. According to Setiaji et al. (2022a, 2023), native rabbit varieties in Indonesia have traditionally been cultivated for a range of uses. To enhance growth performance, there is growing interest in investigating the possible advantages of crossbreeding with imported breeds. According to Setiaji et al. (2022b), farmers have been raising Flemish Giant and Rex animals, two imported rabbit species, for several years. Rex animals have demonstrated excellent reproductive performance, while Flemish Giant rabbits have a superior average daily body weight (BW) gain of 14.99 ± 1.01 g (Gu et al., 2005; Chisowa et al., 2023).

This study explores a crossbreeding experiment involving local Indonesian rabbits and concentrates on a phenomenon known as the “heterosis effect”, in which hybrid offspring display superior traits compared with those of their purebred counterparts. The study aims to enhance understanding of the potential advantages of maximizing production efficiency in a rabbit breeding program by examining the growth performance of crossbred rabbits (García and Argente, 2020; Mondin et al., 2021).

In the context of sustainable agriculture, it is important to comprehend the heterosis effect with respect to Indonesian rabbit breeds, as this will provide breeders, farmers, and policymakers with useful information (Lebas et al., 1997; Lukefahr et al., 2022). This study aims to further the discussion on animal breeding techniques and the possible improvement of local rabbit populations' performance via a thorough analysis of growth-related parameters. In order to evaluate growth performance and growth parameters (estimated by the nonlinear regression model), the study set out to examine the heterosis effect of crossbreeding between two respective imported rabbit breeds and local rabbits.

## 2 Material and methods

### 2.1 Ethical approval

The experimental procedures were approved by the Animal Research Ethics Committee of the Faculty of Animal and Agricultural Sciences, Universitas Diponegoro (no. 59–01/A-01/KEP-FPP).

### 2.2 Animal and data collection

The study used three rabbit breeds: Rex rabbits (R), Flemish Giant rabbits (F), and Indonesian local rabbits (L). In total, ten 12-month-old sires and thirty 7-month-old dams were used in the study. The average BW of L, F, and R animals was 2.16 ± 0.28, 2.82 ± 0.46, and 4.56 ± 0.27, respectively. There were five different types of crosses built, using two sires and six dams in each: two crossbreeds and three pure breeds. The three pure breeds were L (LL), F (FF), and R (RR).

Two crosses, however, were made between the sires of L and the dams of F (LF) and R (LR). Each individual cage held a single rabbit. The entire feed, comprised of a pelletized diet, was given as needed, and an automated nipple system was used to deliver water. The diet's nutritional composition was as follows: 23.46 % fiber, 1.77 % fat, and 19.71 % protein. A total of 169 offspring from five distinct types of crosses were observed until 63 d of age; at 42 d, they were weaned. Every 3 d following birth, each offspring's BW was measured.

### 2.3 Growth analysis

In an initial analysis, the offspring BW data were divided into five types of crosses using a general linear model and the Duncan multiple-range test. The NLIN procedure in the Statistical Analysis System (SAS, 2021) software was utilized to fit the nonlinear logistic model. The model was as follows:

1 $$w\;\left(t\right)=A\;/\;\left(1+B\;x\;\mathrm{exp}\;(-K\;x\;t)\right),\;\;$$

where w(t) is the observed BW of offspring at t days of age (in grams) and t is the animal's age in days. The growth parameters consist of A , the predicted mature live weight of the asymptotic value; B , the turning point of growth; and K , the growth rate constant to achieve an adult weight. Exp is the value of the natural logarithm (2.718). The inflections of the respective age (IA) and weight (IW) were calculated, according to the pattern given in Lupi et al. (2016), as follows:

2 $$\mathrm{IA}=1n\;\left(B\right)/K,$$

and

3 IW=1/2A.

### 2.4 Heterosis effect

The heterosis effect of BW was calculated, using the formula described in Van Vleck (1990), as follows:

4 $$\begin{array}{rl} & \text{heterosis effect of BW}=\\ & \;\frac{\bar{\mathrm{BW}}\;\;\text{of crossbreed}-\left(\frac{1}{2}\sum \bar{\mathrm{BW}}\;\;\text{of pure breed}\right)}{\left(\frac{1}{2}\sum \bar{\mathrm{BW}}\;\;\text{of pure breed}\right)}. \end{array}$$

The heterosis effects of BW were estimated periodically from birth to 63 d of age. After growth analysis, the heterosis effect was also applied to the growth parameters.

## 3 Results and discussion

### 3.1 Results

Table 1 shows the assessment results for five different crossbreeding methods based on the BW from birth to 63 d of age. Among purebred animals, FF displayed the highest BW. At 3, 21, 33, and 45–60 d, RR displayed a comparable BW to FF. LL had a similar birth weight to RR, but LL had a lower BW than RR from 3 to 63 d. The BW of the progeny resulting from the LF cross was generally higher than that of LL but lower than that of FF.

LF and LL had a comparable BW for 6–12 d. At 6–9 d, 18–21 d, and 45–60 d of age, the BW of LF was comparable to that of FF. The LR cross had a birth weight comparable to that of LL and RR. From 3 to 63 d, the BW of LR animals was greater than that of LL animals. The BW of LR animals was comparable to RR animals from 3 to 24 and from 30 to 63 d, but LR rabbits' BW was greater than that of RR animals at 27 d of age.

**Table 1:** The mean ± SE values of offspring body weight for five type of crossbreeds.

Age at	Crossbreed type				
weighing					
	LL	FF	RR	LF	LR
0	44.83 ± 2.52 ${}^{b}$	53.19 ± 1.91 ${}^{a}$	47.44 ± 1.03 ${}^{b}$	43.42 ± 1.29 ${}^{b}$	46.94 ± 1.58 ${}^{b}$
3	53.01 ± 2.53 ${}^{c}$	63.79 ± 2.83 ${}^{a}$	61.22 ± 1.47 ${}^{a}$	54.30 ± 1.57 ${}^{\mathrm{bc}}$	60.03 ± 2.23 ${}^{\mathrm{ab}}$
6	62.74 ± 3.02 ${}^{b}$	82.33 ± 3.16 ${}^{a}$	82.09 ± 1.68 ${}^{a}$	76.47 ± 2.68 ${}^{a}$	81.91 ± 2.75 ${}^{a}$
9	82.48 ± 3.17 ${}^{c}$	98.52 ± 3.68 ${}^{b}$	101.81 ± 1.65 ${}^{\mathrm{ab}}$	100.42 ± 3.70 ${}^{b}$	110.67 ± 3.16 ${}^{a}$
12	108.52 ± 3.38 ${}^{c}$	135.72 ± 4.89 ${}^{a}$	124.78 ± 2.43 ${}^{\mathrm{ab}}$	122.58 ± 4.35 ${}^{b}$	135.30 ± 4.68 ${}^{a}$
15	131.15 ± 4.21 ${}^{c}$	159.34 ± 5.39 ${}^{a}$	153.03 ± 3.64 ${}^{\mathrm{ab}}$	141.75 ± 4.79 ${}^{\mathrm{bc}}$	158.67 ± 5.52 ${}^{a}$
18	137.75 ± 4.69 ${}^{b}$	188.34 ± 8.13 ${}^{a}$	180.69 ± 4.03 ${}^{a}$	168.72 ± 6.89 ${}^{a}$	180.48 ± 6.87 ${}^{a}$
21	157.25 ± 5.20 ${}^{b}$	220.01 ± 10.11 ${}^{a}$	206.09 ± 4.29 ${}^{a}$	204.14 ± 7.45 ${}^{a}$	209.33 ± 8.31 ${}^{a}$
24	182.91 ± 4.14 ${}^{c}$	261.47 ± 8.64 ${}^{a}$	238.56 ± 4.72 ${}^{b}$	232.03 ± 7.70 ${}^{b}$	252.19 ± 8.48 ${}^{\mathrm{ab}}$
27	210.68 ± 4.20 ${}^{c}$	299.34 ± 8.41 ${}^{a}$	276.97 ± 5.79 ${}^{b}$	257.57 ± 6.89 ${}^{b}$	298.83 ± 7.59 ${}^{a}$
30	243.79 ± 6.39 ${}^{c}$	324.69 ± 8.88 ${}^{a}$	309.84 ± 7.33 ${}^{\mathrm{ab}}$	286.02 ± 7.94 ${}^{b}$	329.33 ± 10.4 ${}^{a}$
33	270.01 ± 4.64 ${}^{c}$	372.78 ± 10.5 ${}^{a}$	360.13 ± 8.12 ${}^{a}$	326.01 ± 7.80 ${}^{b}$	371.38 ± 9.90 ${}^{a}$
36	292.68 ± 5.03 ${}^{d}$	408.41 ± 12.1 ${}^{a}$	378.52 ± 9.23 ${}^{\mathrm{bc}}$	359.18 ± 7.81 ${}^{c}$	400.03 ± 9.86 ${}^{\mathrm{ab}}$
39	315.16 ± 5.50 ${}^{d}$	442.91 ± 12.4 ${}^{a}$	402.45 ± 9.90 ${}^{\mathrm{bc}}$	392.85 ± 11.7 ${}^{c}$	432.14 ± 12.4 ${}^{\mathrm{ab}}$
42	350.84 ± 7.03 ${}^{d}$	478.09 ± 12.7 ${}^{a}$	439.17 ± 12.0 ${}^{\mathrm{bc}}$	406.25 ± 11.2 ${}^{c}$	460.17 ± 13.5 ${}^{\mathrm{ab}}$
45	382.39 ± 7.69 ${}^{b}$	489.31 ± 10.7 ${}^{a}$	457.53 ± 14.4 ${}^{a}$	459.19 ± 12.3 ${}^{a}$	475.55 ± 14.9 ${}^{a}$
48	384.01 ± 5.81 ${}^{b}$	531.81 ± 14.3 ${}^{a}$	493.17 ± 16.4 ${}^{a}$	491.55 ± 12.4 ${}^{a}$	509.36 ± 14.5 ${}^{a}$
51	393.94 ± 4.78 ${}^{b}$	572.81 ± 16.1 ${}^{a}$	528.01 ± 19.87 ${}^{a}$	535.64 ± 16.0 ${}^{a}$	538.96 ± 13.2 ${}^{a}$
54	407.82 ± 4.47 ${}^{b}$	617.06 ± 18.2 ${}^{a}$	587.04 ± 18.8 ${}^{a}$	578.9 ± 14.37 ${}^{a}$	569.74 ± 12.0 ${}^{a}$
57	419.29 ± 3.91 ${}^{c}$	662.34 ± 18.2 ${}^{a}$	626.14 ± 18.6 ${}^{\mathrm{ab}}$	621.2 ± 15.67 ${}^{\mathrm{ab}}$	599.37 ± 13.7 ${}^{b}$
60	435.23 ± 4.07 ${}^{c}$	708.78 ± 23.4 ${}^{a}$	661.64 ± 21.11 ${}^{\mathrm{ab}}$	662 ± 17.15 ${}^{\mathrm{ab}}$	627.11 ± 14.8 ${}^{b}$
63	443.88 ± 2.68 ${}^{c}$	764.59 ± 23.3 ${}^{a}$	691.93 ± 23.01 ${}^{b}$	704.97 ± 18.6 ${}^{b}$	657.33 ± 15.5 ${}^{b}$

The following abbreviations are used in the table: LL – purebred cross of Indonesian local rabbits; FF – purebred cross of Flemish Giant rabbits; RR – purebred cross of Rex rabbits; LF – crossbreed between sires of Indonesian local rabbits and dams of Flemish Giant rabbits; LR – crossbreed between sires of Indonesian local rabbits and dams of Rex rabbits.

**Figure 1:**
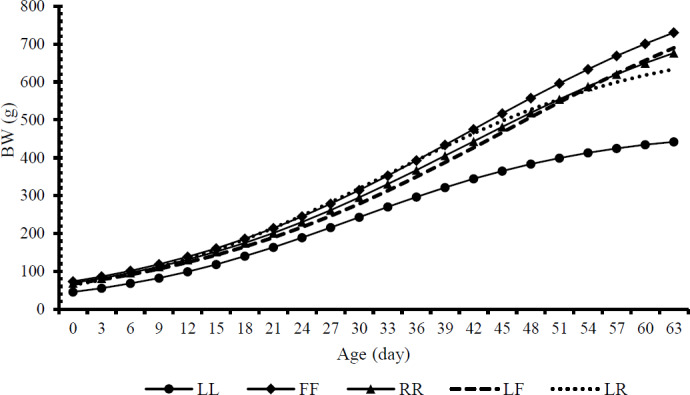
Logistic growth curves fitted to body weight for the offspring of five types of crossbreeds.

The performance of offspring predicted by the logistic model is presented in Fig. 1. The purebred animals with the best performance were identified by the curves as FF, RR, and LL (in that order). Compared with LL rabbits, the predicted performance of crossbred animals was better. Similar to RR, they were directly between FF and LL. When LF rabbits were 48 d of age, their predicted performance was equal to that of RR animals but lower than that of FF and RR. Conversely, LR animals were expected to perform similarly to FF rabbits from 0 to 42 d. After 42 d, its performance declined and became worse than that of FF, RR, and LF.

Table 2 displays the growth parameters estimated by the logistic model. The predicted A for LL rabbits was the lowest within the purebred animals, whereas the A for FF was the highest. RR animals showed slightly lower A values than FF. The crossbred LF rabbits indicated higher A than purebred animals. The A for LR animals was higher than that for LL rabbits, but it was lower than that for RR animals. The same phenomenon was also observed for the value of B . The value of K ranged from 0.06 to 0.08; the highest values were found for LL rabbits, whereas the lowest values was found for LR animals.

**Table 2:** Growth parameters for the offspring estimated using a logistic model.

Growth	Crossbreed type				
parameter					
	LL	FF	RR	LF	LR
A	476.06 ± 7.73	930.43 ± 54.78	858.36 ± 47.09	943.35 ± 53.26	709.81 ± 18.92
B	9.55 ± 0.43	11.75 ± 0.74	11.38 ± 0.67	13.14 ± 0.64	10.18 ± 0.58
K	0.08 ± 0.01	0.06 ± 0.01	0.06 ± 0.01	0.06 ± 0.01	0.07 ± 0.01
IA	29.69	41.76	41.22	45.19	33.15
IW	238.03	465.21	429.18	471.67	354.91

The following abbreviations are used in the table: LL – purebred cross of Indonesian local rabbits; FF – purebred cross of Flemish Giant rabbits; RR – purebred cross of Rex rabbits; LF – crossbreed between sires of Indonesian local rabbits and dams of Flemish Giant rabbits; LR – crossbreed between sires of Indonesian local rabbits and dams of Rex rabbits. The following parameters are listed in the table: A – the predicted mature live weight of the asymptotic value; B – the turning point of growth; K – the growth rate constant to achieve an adult weight; IA – inflections of age; IW – inflections of weight.

The IA ranged from 29.69 to 45.19 d; the IA of LL was the youngest, whereas the IA of FF was the oldest within the purebred animals. LF rabbits showed older IAs than LL and FF animals. The IA of LR animals was younger than that of RR rabbits but older than that of LL animals. LF crossbreeds also showed the heaviest IW compared with purebred rabbits and LR crossbreeds. The IW of LR animals was higher than that for LL rabbits but lower than that for RR animals.

**Figure 2:**
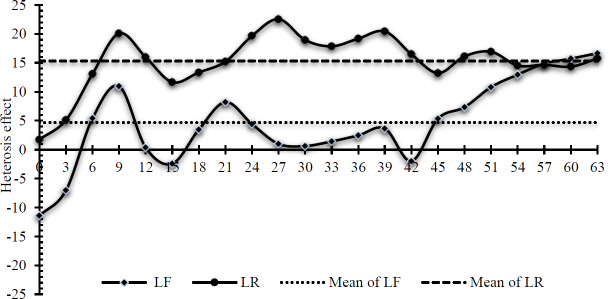
Heterosis effect of crossbreeding for body weight.

The heterosis effects of crossbreeding for BW are presented in Fig. 2. The LF crossbreed's heterosis value varied greatly, ranging from - 11.41 to 16.67. The negative heterosis effect for BW occurred from birth to 5 d, 15 d, and 42 d. The heterosis effect of LF showed a favorable trend after 42 d. The average value of the heterosis effect for LF was 4.68. The LR crossbreed indicated a more stable heterosis effect, ranging from 1.75 to 22.56. The lowest value was at birth, whereas the highest value was at 27 d of age. The heterosis effect was significantly increased from birth to 9 d of age. A sharp decline was observed from 5 to 15 d of age and from 39 to 42 d of age. The same favorable trend as for LF was also shown after 42 d. The average value of the heterosis effect for LR (15.32) was higher than that for LF. Table 3 presents the heterosis effects of crossbreeding for growth parameters. The LF crossbreed indicated a higher effect than LR for the A , B , IW, and IW parameters, whereas LR had a more favorable effect for K . LF rabbits showed a negative heterosis effect for B and IA, whereas LR animals showed that for K .

**Table 3:** Heterosis effects of crossbreeding for growth parameters.

Crossbreed	Growth parameter				
type					
	A	B	K	IA	IW
LF	34.14	23.38	- 15.16	26.48	34.14
LR	6.38	- 2.72	3.71	- 6.51	6.38

The following abbreviations are used in the table: LF – crossbreed between sires of Indonesian local rabbits and dams of Flemish Giant rabbits; LR – crossbreed between sires of Indonesian local rabbits and dams of Rex rabbits. The following parameters are listed in the table: A – the predicted mature live weight of the asymptotic value; B – the turning point of growth; K – the growth rate constant to achieve an adult weight; IA – inflections of age; IW – inflections of weight.

### 3.2 Discussion

Few studies have been conducted on the growth performance and BW of LL animals. Compared with other local breeds from tropical countries, the BW of LL rabbits at 48 d (384.01 g) was lower than that of local Egyptian Baladi rabbits (548 g) (Helal, 2019). The BW values of LL rabbits at 30 d and 60 d were also lower than values reported by Hassan and Al-Barzinji (2022) for local Iraqi rabbits (476 and 953 g, respectively). The growth performance of FF animals in this study was lower than the performance of Flemish Giant animals reported in Europe (Strychalski et al., 2014; Palka et al., 2023). Furthermore, the BW of RR animals in this study was also lower than values reported in China for Rex rabbits (Liu et al., 2018; Chen et al., 2020; Li et al., 2021). Although crossing LF and LR rabbits resulted in better performance than purebred LL animals, it was still not as good as that of purebred FF and RR rabbits. The prospect of crossing LL sires with respective RR and FF dams to increase growth potential, particularly for LL, is based on the dominance of the LF and LR crossbreeds concerning BW. The BW was found to be significantly impacted by genotype, as previously reported by Ouyed et al. (2011). Additionally, Al-Dobaib et al. (2007) observed that crossbred rabbits outperformed their purebred counterparts in terms of growth, indicating that crossing V-line rabbits with Saudi Gabali animals produced higher weights and better increases compared with purebred V-line animals during the 4- to 12-week performance period.

The growth parameters reported using the logistic model for Soviet Chinchilla and White Giant rabbits were higher than the estimated values of A , B , and K in this study (ranges of 476.06–943.35, 9.55–13.14, and 0.06–0.08, respectively) (Gupta et al., 2022). Comparing the estimated IA and IW for LL and FF animals, using the Gompertz model reported by Setiaji et al. (2013), revealed that the estimates for FF animals were lower (41.76 and 465.21 g, respectively), whereas the estimates for LL rabbits were slightly higher (29.69 and 238.03 g, respectively). While LR displayed higher growth parameters and inflection points than LL animals but lower values than RR animals, the estimated growth parameters and inflection points for the LF crossbreed were superior to those of purebred LL and FF animals.

Compared with the LR crossbreed, the heterosis effect for BW for the LF crossbreed was smaller. It is observed that the LR crossbreed exhibits a stronger heterosis effect than the LF crossbreed, as indicated by improved growth performance. Rex rabbits add special genetic qualities to the crossbreeding equation. They are distinguished by their plush fur and distinctive coat colors. Conversely, Flemish Giant rabbits are renowned for their substantial size and remarkable growth rates. A higher heterosis effect indicates that offspring with more pronounced and advantageous growth performance traits are being produced by combining genetic material from local rabbit and Rex breeds (Prayaga and Eady, 2003). On the other hand, based on the growth parameter and inflection point determined by the logistic model, the LF crossbreed demonstrated better heterosis effects. This example may be caused by the nonlinear growth observed in scenarios in which competition, resource limitations, or saturation points influence growth and lead to more complex pathways (Friggens et al., 2017).

## 4 Conclusions

This research adds to the growing body of knowledge on the growth dynamics and heterosis effects in various crossbreeding scenarios involving the local Indonesian rabbit breed and improves rabbit breeding programs with respect to higher productivity.

## Data Availability

The data used and analyzed during this study are available from the corresponding author upon reasonable request.

## Code availability

The underlying software code used during this study is available from the corresponding author upon reasonable request.
